# Epidemiology and eradication of infectious bovine rhinotracheitis/infectious pustular vulvovaginitis (IBR/IPV) virus in Finland

**DOI:** 10.1186/1751-0147-49-3

**Published:** 2007-01-12

**Authors:** Lasse Nuotio, Erkki Neuvonen, Mauno Hyytiäinen

**Affiliations:** 1 Department of Virology, National Veterinary and Food Research Institute EELA, PO Box 45, FI-00581 Helsinki, Finland; 2 retired from State Provincial Office of Southern Finland, P.O. Box 150, 13101 Hämeenlinna, Finland

## Abstract

**Background:**

Infectious bovine rhinotracheitis/infectious pustular vulvovaginitis (IBR/IPV) is a significant disease among domestic and wild cattle. The BHV-1 infection was first detected in Finland in 1970; presumably it was imported in 1968. The infection reappeared in the large-scale bulk-tank milk surveillances which started in 1990, and was eradicated in 1994. Our aim is to describe the epidemiology of this infection in Finland, and its eradication.

**Materials and methods:**

The official sources of pertinent information, the legal basis for the disease control and the serological methods for the detection of the infection are described.

**Results and conclusion:**

Ten AI bulls were found to be seropositive in 1970–1971. The total number of herds with BHV-1 antibody positive animals in the large-scale surveillance in 1990 and subsequent epidemiological investigations in 1991 was five, and the total number of seropositive animals was 90. The five herds formed three epidemiological units; semen of at least one bull seropositive in 1971 had been used in each unit. This remained the only plausible route of infection in each of the three units. Using the 'test and slaughter' approach and total stamping out in one herd the infection was eradicated in 1994.

## Background

Infectious bovine rhinotracheitis/infectious pustular vulvovaginitis (IBR/IPV) is a significant disease among domestic and wild bovine animals [1,2]. Clinical signs of the infection include symptoms of inflammatory processes on both respiratory and genital organs, and abortion. Young calves can develop a systemic disease affecting visceral organs [3].

The disease is caused by the bovine herpesvirus 1 (BHV-1), in the subfamily Alphaherpesvirinae of the Herpesviridae family. Only a single serotype of BHV-1 is recognized, but subtypes of BHV-1 have been described on the basis of restriction enzyme cleavage patterns of viral DNA. These types are referred to as BHV-1.1 (respiratory subtype), BHV-1.2 (respiratory and genital subtype) [4]. BHV-1 is able to establish a latent infection in the trigeminal or sacral ganglia [5]. Animals with a latent BHV-1 infection may serve as a source of infection for susceptible animals if and when the virus is reactivated [6]. The infection is transmitted mainly through respiratory, ocular or genital secretions in a direct contact between animals. However, the infection can also spread through fresh or frozen semen from infected bulls [7-9].

The disease is notifiable in many, but not all countries. Common control measures against IBR/IPV include screening, surveillance, precautions at borders and a modified stamping out policy. Many countries allow and carry out vaccinations, while vaccination is prohibited in countries that have eradicated the disease [2]. Serological testing for the infection in Finland started in 1965, and antibodies against BHV-1 were encountered for the first time in 1970–1971 among bulls at an artificial insemination (AI) station. The infection surfaced again in 1990 in a large herd with respiratory problems among calves, and in the first bulk-tank milk (BTM) surveillance for selected viral infections in the same year. The infection was eradicated in 1994. Our purpose is to review the official control measures, the epidemiology of the infection in Finland and its eradication.

## Materials and methods

### Sources of the data

The data of the targeted testing of specific groups of animals for antibodies against BHV-1 between 1965 and 1989 were extracted from the annual statistics compiled by the State Veterinary Institute. The tested animals consisted mainly of heifers to be exported to the Soviet Union, live animals imported into Finland, and animals with respiratory infections for which a definite diagnosis was considered necessary. The sample was not actually random but selected, taken each year, especially in the case of heifers, from the same BHV-1 free herds. The data are shown in Table 1. Systematic testing of sera from AI bulls was introduced in 1978.

**Table 1:** Mean, standard deviation (SD), range and number of positive results of number of serum samples tested for antibodies against BHV-1 in 1965–1989

Period	No of samples examined annually			
	
	Mean	SD	Range	Nr positives
1965 – 1973	68	54	4 – 159	10^1^
1974 – 1985	661	158	335 – 890	0
1986 – 1989	2126	1126	523 – 2947	0

1: AI bulls at a station in 1970–1971

Each dairy herd was tested annually since 1991 for antibodies against BHV-1. The individual serum samples in Table 2 represent the continued targeted testing of specific groups of animals after 1989. Besides this, a random sample of sera of beef animals were tested since 1993 (see Table 2). The numbers were extracted from the annual statistics compiled by the State Veterinary Institute and, since 1994, by National Veterinary and Food Institute. In 1990 the tested dairy herds were a random sample, approximately 1/4 of the total. Every 3^th ^to every 5^th ^of the beef animals were sampled at the slaughterhouse, when the daily number of the animals to be slaughtered allowed this, otherwise every animal was sampled. The range of the proportion of beef herds these samples represented was estimated to be 70–90%. The insemination statistics of the implicated bulls, and of the herds and were obtained from the registers of ProAgria Agricultural Data Processing Centre Ltd, Vantaa Finland, the latter with written permission from the herd owners.

**Table 2:** BTM surveillance and testing of individual sera for antibodies agains BHV-1 in 1990–2003

Year	No of bulk-tank milk samples	No positive	No of individual serum samples	No of new positive	No of serum samples from beef animals	No positive
1990	9879^(a^	3	2420	0	-	-
1991	36899	1^(b^	6224	90	-	-
1992	37923	1^(b^	7496	0	-	-
1993	34115	1^(b^	4954	0	3248	0
1994	34169	1^(b^	5210	0	12764	0
1995	32588	0	3078	0	2544	0
1996	30569	0	2343	0	2839	0
1997	28577	0	2903	0	2845	0
1998	26934	0	2125	0	2758	0
1999	24872	0	2296	0	2920	0
2000	22698	0	2688	0	2899	0
2001	21040	0	2407	0	2996	0
2002	19870	0	2021	0	2816	0
2003	18519	0	2321	0	6753	0

(a: A sample, 1/4 of the total herds

(b: Same herd, slaughtered in 1994

### Finnish legislation concerning IBR/IPV

An administrative decision was issued by the Ministry of Agriculture and Forestry (MAF) in 1978 which required systematic testing of AI bulls for antibodies against BHV-1. A circular was sent out by MAF in 1993 defining the measures for controlling IBR/IPV, based on the amended Act (809/92) to the Animal Diseases Act (55/80). The disease is notifiable, and a herd suspected of having animals with IBR/IPV is placed under restrictive measures by the official municipal veterinarian. The decision to take restrictive measures can also be based on results from the annual BTM surveillance. All animals in a suspect herd are examined serologically for antibodies against BHV-1. If the results are negative the restrictions are withdrawn.

The herd owner is informed in writing of the decision to take restrictive measures and these include the following:

- All seropositive and clinically ill animals must be isolated to the extent possible and kept indoors

- Bovine animals can only be transported from the farm for slaughter

- Delivery of bovine semen from the herd and the use of animals for natural mating are prohibited

- After the seropositive animals have been culled the stalls and appliances must be cleaned and disinfected with disinfectants that are active against viruses

- Unnecessary visits to the animal shed must be avoided. The use of protective clothing and footwear in the animal shed is mandatory

- No restrictions are imposed on the use of milk or its delivery to the dairy.

Restrictive measures are only withdrawn after seropositive animals have been culled and the other animals have tested negative twice, one month after the culling and then four months after the first test, at the earliest. The slaughter can be carried out at partial state expense (75% of the animal's value with the slaughter value deducted) on application by the owner and recommendation by the municipal and district veterinarians. The municipal veterinarian is obliged to conduct an epidemiological investigation to map the spread of the infection. Vaccination against IBR/IPV in Finland is subject to approval by the Food and Health Department of the Ministry of Agriculture and Forestry.

### Serological methods to detect IBR/IPV

The serological method used in testing prior to 1990 was virus neutralization (VN) test, also referred to as serum neutralization (SN) test. This is a prescribed test for international trade [1], but the actual sensitivity (se) or specificity (sp) of the test depend on laboratory performance. The se or sp of the pre-1990 test method performed in State Veterinary Institute (VELL, later EELA) can no longer be traced. The VN test has only been used since 1990 for confirmatory purposes. The commercial ELISA kit, CHEKIT-Trachitest (Dr Bommeli AG, CH-3097 Liebefeld-Bern, Switzerland) has been used to test individual animal sera since 1990. The same test was also used in 1990–1994 for large scale annual surveillance for antibodies in BTM samples, but the SVANOVA IBR-Ab Kit (SVANOVA Biotech AB, Uppsala Science Park, SE-751 83, Sweden) replaced it in 1995. Estimates of the se or sp for the CHEKIT-Trachitest are not available from the manufacturer, but Rosskopf et al. [10] evaluated the test with material from herds with a known disease status. According to the report, the CHEKIT-Trachitest showed a sensitivity of 94–99.3% and a specificity of 81.5–98.5% for serum samples. The manufacturer of the SVANOVA IBR-Ab kit quotes figures of 100% se and 92% sp, relative to SN test. The se and sp of milk vs. serum are quoted as 92.8% and 100%, respectively. Kits without a control well for each sample were used in large scale testing for reasons of economy. Positive samples were retested using kits with control wells.

## Results

### Surveillance of the infection

The numbers of sera examined for antibodies against BHV-1 in the annual targeted testing prior to 1990, as well as the results of the serological testing are shown in Table 1. The increase in the numbers reflects revisions of administrative decisions concerning animal disease control.

Data of the annual BTM surveillance and of targeted testing of both routine disease testing samples and random samples from beef herds, sent into the national central laboratory between 1990 and 2003, are shown in Table 2

### Epidemiological information on BHV-1 antibody-positive animals and herds

The infection was encountered for the first time in December 1970, when the serum of an AI bull showing signs of respiratory infection tested positive for antibodies against BHV-1. Nine of its AI station mates were also found to be seropositive on closer examination in 1971 (Table 1). One of the seropositive bulls had been imported from Denmark in 1968 and it is quite possible that this animal brought the infection to the station and spread it to the other bulls. Importing of live animals to AI stations required a permit in the sixties, but was still possible. The total number of antibody-positive herds detected in 1990–1991 was five (Table 3): a cluster of four herds in southern Finland, and one separate herd in the north-western part of the country. The total number of traced contact herds of these five herds between 1988 and 1991 was 54. All individual animals of the herds B, D and F, and the contact herds C and E, except of one non-compliant herd, were examined in 1991. All 28 contact herds of herds C and E were also traced and their animals tested individually. All except D, contact of E, were seronegative. Details of the AI station, the five seropositive herds, and epidemiological information are presented in Table 3.

**Table 3:** Information on the AI station and herds containing BHV-1 antibody-positive animals

Herd (station)	Type of production	Apprx nr animals	Nr seropos. animals	Year herd detected	Epidemiol. connection to	Type of contact	Cause of detection
A	AI station	30	10	1970	abroad	import^1^	Clinical suspicion
B	Dairy and beef	450	79	1990	A	s^2^	Clinical suspicion
C	Dairy	25	1	1991	B	p, a^3^	Contact herd of B
D	Dairy	50	6	1990	E	a^4^	BTM antibody pos.
E	Beef, calf rearing	55	2	1991	A, D	s	Contact herd of D
F	Dairy	15	2	1990	A	s	BTM antibody pos.

1: see text for details

2: possibly contaminated semen

3: same cattle tender working both in B and C; animal

4: animal

The capital letter denotation of herds in the following refers to Table 3. Herd B was detected by signs of respiratory infection, such as fever, nasal discharge and dyspnea in young stock; however, herd B was also BTM antibody-positive; this is reflected in Table 2 where the number of BTM antibody-positive herds in 1990 is three. The herds formed three apparent epidemiological units. Herds B and C were neighbours and had animal and personnel contacts. The only seropositive animal in herd C had been purchased from B. Herd E served as a young stock raising operation for herd D, such that the heifers were inseminated when in herd E and returned to herd D approximately 1 month before parturition. The third epidemiological unit was the geographically separate herd F. The insemination statistics of the herds revealed that semen from three AI bulls found to be seropositive in 1971 had been used in herds B, E and F, and had come from a different bull for each herd. This means that possibly infected semen had been used in each epidemiological unit, which remains the only plausible route of transmission for each unit.

### Applied control measures

The seropositive bulls on the AI station were not culled as this was not required by law, and many served the full term. The herd B and the BTM antibody-positive herds D and F were placed under restrictive measures in 1991. The seropositive animals in herds D and F were culled and both the BTM and individual sera of the animals in these herds were retested after the required periods. In both cases the subsequent samples were all negative and the restrictive measures were withdrawn. The seropositive contact herds C and E were dealt with in the same way as herds D and F. The one non-compliant contact herd (of herd B) was also placed under restrictive measures, as a suspected BHV-1 positive herd. The two animals bought from herd B to this herd were tested at slaughter and found to be seronegative. The herd was subsequently cleared in 1993. After four years under restrictive measures the herd B was entirely stamped out in 1994.

## Discussion

The rarity of the BHV-1 infection in Finland indicates an introduction in the late sixties, despite the fact that herd management practices do not in general favour the spread of infectious agents. The infection was not mentioned in the comprehensive review of animal virus diseases in Finland, published in 1964 [11]. It is quite possible that the semen from the three implicated AI bulls, that was used between 1968 and 1979 spread the infection to a number of dairy herds and that it persisted in these herds for over a decade. E.g. Mollema et al. [12] have shown that herpesvirus can persist for more than 40 years in small (n = 20) host populations. The average herd size was around 10 in the sixties and is still less than 20 in 2006. Another conceivable route for the introduction of BHV-1 into the country could have been through the extensive contacts with foreign personnel on one of the herds (herd B). However, there is nothing to substantiate this claim and it could only explain the infection for one of the epidemiological units. Unfortunately no isolates of the virus were ever obtained from the semen or the infected animals to provide sequence-based phylogenetic support for a common origin. Thus there is no conclusive proof of the semen-mediated transmission, however, taken together the well known potential of transmitting IBR via semen, the fact that semen from infected bulls had been used in each of the epidemiological units and the very strict official import policy on live animals before 1995, this route is by far the most probable.

A figure for the total number of supposedly semen-infected dairy herds can be estimated from the proportion of known infected herds (3) to the total number of dairy herds in 1990 (45,500), and the total number of dairy herds which used AI services in early seventies (120,000) [13], assuming that semen was indeed the only source of infection and that the number of infected herds had only been reduced by the closure of dairy farms. When you divide the estimated 8 herds into the total number of herds (23,820) in which semen from the three implicated AI bulls had been used, then this gives a transmission efficacy of approximately 1/3,000 inseminations. The low efficacy concurs with the notion that the shedding of the virus into the semen only takes place for a few days [14], or is intermittent and that the titer of the virus in semen is only occasionally sufficient to infect the dam [15,16].

Routine annual screening between 1972 and 1989, including AI bulls, did not reveal any BHV-1 antibody-positive animals. This is not surprising given that the mean number of samples examined between 1986 and 1989 (Table 1) represented only 0.15% of the contemporary cattle population of approximately 1,400,000 heads. The probability of detecting an infection with this sample size, using the total number of seropositive animals (90) as an estimate for the infection prevalence (0.006%), is no more than 12% (assuming a random sample and a perfect test; calculated with WinEpiscope 2.0, see [17] for details).

IBR/IPV has been eradicated during the nineties from a few European countries, e.g. Austria, Denmark and Sweden, and the disease-free status of these countries was last confirmed by the EU Commission in 2004 [1]. Switzerland and Norway are also recognized as being free of the disease [18,19]. These countries do not vaccinate against IBR/IPV. The role of vaccination in eradication campaigns is controversial. Kujik [20] claimed that if there are > 15–20% seropositive animals in the population, vaccination is the most realistic strategy to eradicate IBR/IPV. Noordegraaf et al. [21] even submitted the idea that a compulsory vaccination programme would be necessary to eradicate the disease. On the other hand, separation or culling of the seropositive animals and restocking with seronegative animals is shown to be a valid alternative to vaccination, at least at the herd level [22,23]. In Finland the clinical suspicion, BTM antibody surveillance and epidemiological investigations in 1990–1991 rounded up five BHV-1 antibody-positive herds. Using vaccines to control and eradicate the infection was deliberated (L. Sihvonen, personal communication) but there were no marker vaccines available at the time and the situation was not considered serious enough to justify the risks involved in using live vaccines. Furthermore, the small total number of known infected herds did not support the notion of a wholesale vaccination campaign. Hence a decisive 'test and slaughter' policy to control IBR/IPV was adopted. This policy proved successful, which is reflected by the short time, i.e. 5 years, between the detection and eradication of the infection. The time could have been even shorter, had it not been for one herd (Table 2). Cattle owners were entitled to financial compensation if animals from their herds were culled for seropositivity or the actual disease and this was no doubt a key to the success of the project, as was the case with the eradication of enzootic bovine leukosis [24].

Annual surveillance of all dairy herds for antibodies against BHV-1 in BTM samples and of beef animals at slaughter are continuing and the results are reported to the EU Commission. The Commission set additional guarantees relating to IBR/IPV for bovines destined for Finland in 1994 based on the country's disease free status, and these guarantees were last confirmed in 2004 [18].

## Conclusion

The available information suggests that the BHV-1 virus infection was imported into Finland in 1968 along an infected AI bull, and that it spread in 1968–1979 via semen of the bulls that contracted the infection at the station. Once the extent of the spread was established in large-scale annual surveillances in 1990–1992, effective control measures at the expense of the state were taken. The infection was eradicated in 1994 and the additional guarantees of EU Commission have applied to Finland since then. Annual bulk-tank milk surveillances and testing of individual animal sera for antibodies against BHV-1 are continuing

## Competing interests

The authors declare that they have no competing interests.

## Authors' contributions

LN and EN participated equally in the design of the study. LN compiled the annual disease monitoring statistics and insemination information. EN and MH compiled the data of the farm and provincial level epidemiological investigations. LN drafted the manuscript and the other authors read and approved the final manuscript.
